# Mutual interaction between endoplasmic reticulum and mitochondria in nonalcoholic fatty liver disease

**DOI:** 10.1186/s12944-020-01210-0

**Published:** 2020-04-13

**Authors:** Jin Wang, Wanping He, Ping-Ju Tsai, Pei-Hsuan Chen, Manxiang Ye, Jiao Guo, Zhengquan Su

**Affiliations:** 1 Guangdong Engineering Research Center of Natural Products and New Drugs, Guangdong Provincial University Engineering Technology Research Center of Natural Products and Drugs, Guangdong Pharmaceutical University, Guangzhou, 510006 China; 2 Guangdong Metabolic Diseases Research Centre of Integrated Chinese and Western Medicine, Guangdong TCM Key Laboratory for Metabolic Diseases, Key Laboratory of Modulating Liver to Treat Hyperlipemia SATCM, Level 3 Laboratory of Lipid Metabolism SATCM, Institute of Chinese Medicinal Sciences, Guangdong Pharmaceutical University, Guangzhou, 510006 China; 3 King-Prebiotics Biotechnology (TW) Co., LTD, 2F.-1, No. 250, Zhongshan Rd., Linkou Dist, New Taipei City, 24446 Taiwan; 4 New Francisco (Yunfu City) Biotechnology Co, Ltd Swan-kan-chiau Ind. Dist., Kaofong Village, Yunfu City, Guangdong China

**Keywords:** Endoplasmic reticulum stress, Non-alcoholic fatty liver disease, Mitochondria-associated membrane, Oxidative stress, Calcium ion homeostasis

## Abstract

Nonalcoholic fatty liver disease (NAFLD) is a common metabolic syndrome. Imbalances between liver lipid output and input are the direct causes of NAFLD, and hepatic steatosis is the pathological premise and basis for NAFLD progression. Mutual interaction between endoplasmic reticulum stress (ERS) and oxidative stress play important roles in NAFLD pathogenesis. Notably, mitochondria-associated membranes (MAMs) act as a structural bridges for functional clustering of molecules, particularly for Ca^2+^, lipids, and reactive oxygen species (ROS) exchange. Previous studies have examined the crucial roles of ERS and ROS in NAFLD and have shown that MAM structural and functional integrity determines normal ER- mitochondria communication. Upon disruption of MAM integrity, miscommunication directly or indirectly causes imbalances in Ca2+ homeostasis and increases ERS and oxidative stress. Here, we emphasize the involvement of MAMs in glucose and lipid metabolism, chronic inflammation and insulin resistance in NAFLD and summarize MAM-targeting drugs and compounds, most of which achieve their therapeutic or ameliorative effects on NAFLD by improving MAM integrity. Therefore, targeting MAMs may be a viable strategy for NAFLD treatment. This review provides new ideas and key points for basic NAFLD research and drug development centred on mitochondria and the endoplasmic reticulum.

## Introduction

**Fig. 1:**
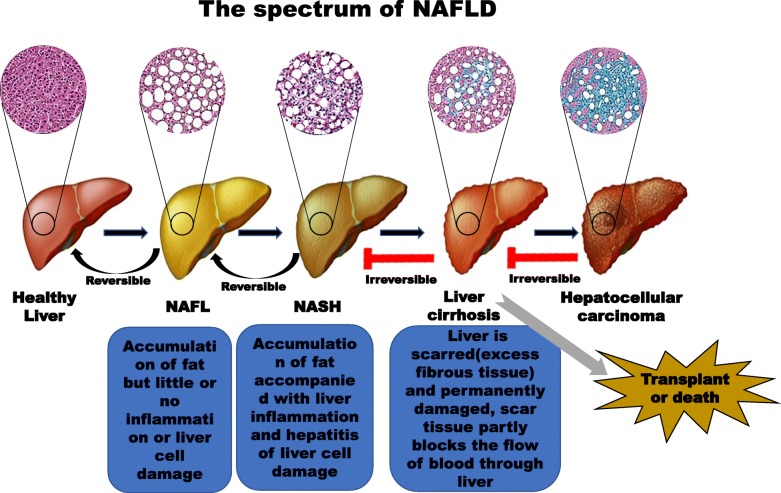
Spectrum of NAFLD progression. The development of NAFLD is divided into four stages: simple steatosis (or NAFL), NASH, liver cirrhosis, and eventually HCC [1]. Factors that cause simple steatosis include Western HFHSDs, obesity, T2DM (particularly the associated IR), and other metabolic diseases. Factors that contribute to the development of NASH include inflammation and hepatocyte apoptosis. Liver fibrosis is a transitional phase of NASH that results in the development of liver cirrhosis. Liver fibrosis is divided into four stages according to the degree of fibrosis present in patients with NASH and cirrhosis

Nonalcoholic fatty liver disease (NAFLD) is a spectrum of liver disorders caused by fat accumulation in the liver (steatosis) due to factors other than excessive alcohol use (Fig. 1) [2]. NAFLD is divided into two major subtypes: nonalcoholic fatty liver (NAFL also called simple steatosis), which is a form of nonprogressive NAFLD that rarely develops into cirrhosis, and nonalcoholic steatohepatitis (NASH), a progressive and aggressive form of NAFLD. Long-term NASH is highly likely to progress to cirrhosis and hepatocellular carcinoma (HCC) [1]. NASH is considered an intermediate stage of liver damage and the most extreme form of NAFLD [2]. Studies have shown that obesity is a major risk factor for insulin resistance (IR), and IR is a key factor in NAFLD. In turn, IR promotes the occurrence of metabolic syndrome, type 2 diabetes mellitus (T2DM), and NAFL. IR also promotes the development of NASH and even liver fibrosis. Therefore, most patients with NAFLD usually also exhibit diseases such as obesity, IR, hypertension and hyperlipidaemia [3, 4]. There is still much controversy as to whether NAFLD/NASH is a primary or secondary disease related to obesity and T2DM. The global prevalence of NAFLD is increasing, and NAFLD will soon become a major cause of liver-related morbidity in many countries and regions. The epidemiological and demographic characteristics of patients with NAFLD are globally diverse [5]. Due to differences in diet and lifestyle, the prevalence of NAFLD is significantly higher in Western countries (20–30%) than in Eastern countries (10–20%). Obesity, T2DM, hyperlipidaemia, hypertension, and metabolic syndrome are major risk factors for NAFLD and are quite common in Western industrialized countries [6]. The incidence of NAFLD in patients with T2DM and patients with obesity ranges from 40 to 80% and from 30 to 90%, respectively [7]. More than 90% of people with severe obesity have NAFLD [8].

In addition to liver parenchymal cells, which play decisive roles, liver non-parenchymal cells can also affect the development of NAFLD. Liver non-parenchymal cells include hepatic stellate cells (HSCs), macrophages (Kupffer cells), pit cells, dendritic cells, and liver sinusoidal endothelial cells (LSECs). During NAFLD, these cells may mediate liver inflammation and fibrosis due to long-term liver lipotoxicity and hepatocyte damage [9]. Kupffer cells are resident macrophages in the liver and include classic M1 type (pro-inflammatory) cells and alternative M2 type (anti-inflammatory) cells. A relative balance between M1 and M2 cells helps repair and reshape damaged liver tissue [10]. Palmitate promotes Kupffer cells to polarize to a pro-inflammatory M1 phenotype, while polyunsaturated fatty acids (PUFAs) increases anti-inflammatory M2 macrophage populations. Hepatic palmitate content generally increases during the progression of NAFLD, which leads to an imbalance in the M1/M2 type ratio and accelerates liver inflammation [11]. HSCs are distributed around the sinuses throughout the liver. Under normal circumstances, HSCs are at rest. However, when the liver is damaged by inflammation or mechanical stimulation, HSCs are activated and transformed into myofibroblast-like cells (MFCs) [12]. HSCs are the ultimate target cells for various fibrogenic factors. HSCs can also be activated in response to inflammation and hepatocyte damage caused by lipid accumulation or lipotoxicity during NAFLD [9, 12]. Notably, LSECs are the most abundant non-parenchymal cells in the liver. Lipid accumulation during long-term NAFLD development causes LSEC lipotoxicity [9], but little is known about the contribution of LSEC lipotoxicity to the progression of NAFLD. Under normal circumstances, LSECs mainly maintain the stability of liver vascular tension by producing nitric oxide, thereby keeping HSCs stationary under steady-state conditions. LSEC lipotoxicity leads to decreases in both nitric oxide and reactive oxygen species (ROS) levels, resulting in oxidative stress in LSECs, and LSEC-derived ROS can enter the circulatory system and promote the NAFLD process [13]. One study also found elevated ratios of neutrophils to lymphocytes in patients with NASH and NASH-related liver fibrosis [14].

**Fig. 2:**
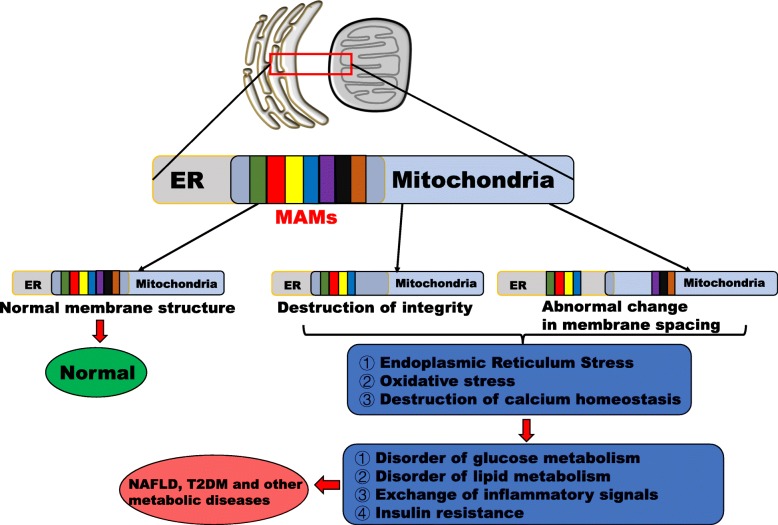
Pathological states and associated hepatic metabolic diseases resulting from disruption of the structural and functional integrity of MAMs. Mitochondria and the ER intersect functionally and structurally in areas that researchers call MAMs. Many mitochondrial and ER-related proteins or signalling molecules are located here. The structural and functional integrity of MAMs determines the normal communication between the ER and mitochondria. Damage to the integrity of MAMs, including changes in the levels of membrane proteins (increases or decreases) and suboptimal spacing of MAMs (too close or too far), can lead to miscommunication between the organelles, which can directly or indirectly lead to hepatic ERS, oxidative stress and imbalanced Ca^2+^ homeostasis. When these hepatocyte problems are amplified, glucose and lipid metabolism disturbances, chronic inflammation and IR develop. T2DM, NAFLD, and other metabolic diseases occur or develop when mechanisms in the body are insufficient to counteract these dangerous pathological reactions. Therefore, ER–mitochondria miscommunication is involved in the pathophysiology of T2DM and NAFLD

Both organelles and mitochondria-associated endoplasmic reticulum (ER) membranes (MAMs) play crucial roles in sensing hepatocyte nutrient levels and energy metabolism, particularly with regard to the exchange of substances required for metabolic homeostasis, such as metabolites, ROS and Ca2+. These organelles, physiological responses and signalling substances play decisive roles in the occurrence and development of NAFLD [15]. However, hepatocytes often exhibit disturbances in Ca2+, energy metabolism and redox homeostasis, which may be accompanied by hepatocyte signalling disorder, inflammation, apoptosis and death in patients with NAFLD [16]. In particular, oxidative stress and ER stress (ERS) have become important biochemical markers of NAFLD. Most researchers tend to separate the two processes to study their contributions to NAFLD, but the maintenance of homeostasis between mitochondria and the ER is interdependent to a certain extent. In this review, the roles of the ER and mitochondria and the interactions between the two during the development of NAFLD will be discussed. These interactions include regulation of Ca2+ homeostasis and glycolipid metabolism by MAMs, ERS, ROS production and oxidative stress during NAFLD (Fig. 2). There are few reports on the contributions of MAMs to the progression of NAFLD in liver non-parenchymal cells. It is possible that non-parenchymal cells maintain normal functions and structures in the early stages of NAFLD. When the liver is sufficiently stimulated, the functions and structures of these cells may change, affecting the development of NAFLD.

## Pathogenic and molecular mechanisms of NAFLD

### Pathogenesis of NAFLD and glycolipid metabolism

Theoretically, dietary carbohydrates and lipids and lipids produced through de novo lipogenesis (DNL) have been considered essential contributors to triglyceride (TG) synthesis in the human liver. TG accumulation is the initial step in the development of the pathophysiology of NAFLD and is caused by an imbalance between TG synthesis and degradation [17].

The liver can take up free fatty acids (FFAs) from plasma to synthesize TG. Under fasting conditions, beta-adrenergic receptor agonists induce lipolysis to produce FFAs. In the case of IR in patients with obesity, decomposition of large amounts of fat increases the concentrations of FFAs in plasma [18]. As transmembrane proteins, fatty acid (FA) transporter protein (FATP), caveolins, FA translocase (FAT)/CD36, and FA binding protein (FABP) can accelerate FA uptake by promoting FFA proliferation in blood vessels, and a high-fat, high-sugar diet (HFHSD) increases the expression of these proteins [19, 20]. In particular, when liver TG levels increase, the expression of CD36 increases in patients with NAFLD [21].

**Fig. 3:**
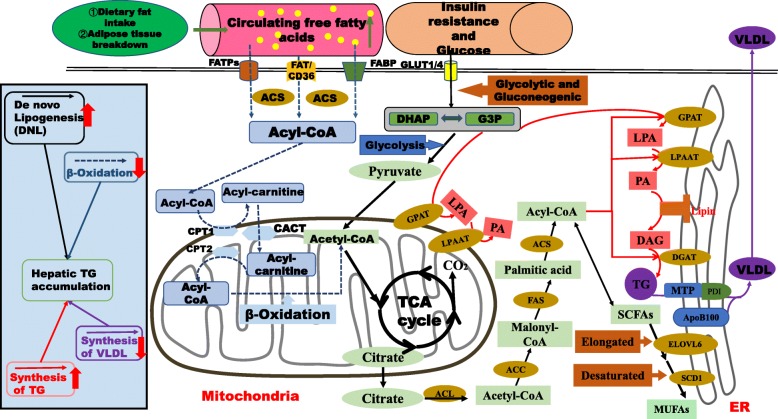
Liver lipid synthesis, accumulation and oxidation. Sources of liver fat: Dietary consumption of FAs, adipose tissue breakdown to produce FFAs, and DNL. FFAs are ultimately processed into TGs and stored as fat droplets in the liver. DNL: Palmitic acid and FAs are synthesized from glucose under the catalysis of various enzymes. ACS catalyses the formation of palmitoyl-CoA from PA via acetyl-CoA, which is extended and desaturated, respectively, under the action of ELOVL6 and SCD1. Finally, MUFAs are generated. TG synthesis: G3P and acyl-CoA synthesis by DNL is catalysed by GPAT to generate LPA. With catalysis of LPAAT, PA is generated by acylation of LPA with another acyl-CoA. PA is dephosphorylated under the catalysis of PAP to produce DAG. Finally, DAG is acylated to form TG by an acyl-CoA molecule under the catalytic activity of DGAT. FA oxidation: Acyl-CoA molecules are transported into the mitochondria by the activity of CPT1, CPT2 and CACT; the acyl-CoA molecules are then oxidized to form acetyl-CoA. VLDL synthesis: In hepatocytes, TG, cholesterol and APOB100 combine in the ER via MTP activity to form VLDL, which is released into the blood. In patients with NAFLD, hepatic steatosis can be stimulated via increased FA uptake, increased DNL, decreased VLDL secretion and decreased FA β-oxidation followed by esterification for TG synthesis

DNL is the process by which lipids are synthesized from exogenous carbohydrates or endogenous energy sources. This process involves three consecutive steps: the synthesis of FAs from acetyl-CoA subunits produced during glycolysis and carbohydrate metabolism, FA elongation and desaturation to form long-chain unsaturated FAs, and assembly of the FAs into TGs and very low-density lipoproteins (VLDLs) [22] (Fig. 3). The final pyruvate product formed from carbohydrates in the cytoplasm is transported into the mitochondria and converted into acetyl-CoA for participation in the tricarboxylic acid (TCA) cycle for energy supply. When the supply and storage of energy reach saturation, the citrate intermediate in the TCA cycle begins to accumulate in large amounts and enters the cytoplasm to participate in lipid synthesis [17]. Glucokinase and liver-type pyruvate kinase convert glucose to acetyl-CoA during glycolysis. The resulting acetyl-CoA molecules are catalysed by acetyl-CoA carboxylase (ACC) to form malonyl-CoA. The malonyl/acetyltransferase (MAT) site and the thioesterase (TE) domain of FAS are activated sequentially to catalyse the release of palmitic or stearic acid from ACP [23]. At the beginning of the synthesis of triacylglycerol (TAG), glyceraldehyde 3-phosphate (G3P) and acyl-CoA synthesis by DNL is catalysed by glycerol-3-phosphate acyl transferase (GPAT) to generate lysophosphatidic acid (LPA) [24]. With catalysis of LPA acyltransferase (LPAAT), phosphatidic acid (PA) is generated by acylation of LPA with another acyl-CoA. PA is dephosphorylated under the catalysis of PA phosphorylase (PAP) to produce diacylglycerol (DAG). Finally, DAG is acylated to TG by an acyl-CoA molecule under the catalytic activity of diacylglycerol acyltransferase (DGAT) [25] (Fig. 3).

The main site of cholesterol synthesis is the liver, and acetyl-CoA is catalysed by thiolytic enzymes to condense acetoacetyl-CoA, which generates HMG-CoA under the action of monoacyl-CoA (HMG-CoA) synthetase. HMG-CoA reductase promotes cholesterol synthesis as a rate-limiting enzyme [26]. Cholesterol is exchanged and transported through cholesterol ester transfer protein (CETP), while the remaining phospholipids, ApoE and ApoC, in VLDL are transferred to high-density lipoprotein (HDL), and VLDL is transformed into VLDL residues. Most of these residues are then integrated into the liver through the VLDL receptor, while a few are converted into low-density lipoprotein (LDL) to continue metabolism [27]. A total of 65–70% of LDL in plasma is cleared by LDL receptors, and a small proportion (approximately 1/3) is taken up by the surrounding tissues (including the liver) and dissimilated. Most VLDL residues are converted to LDL and recognized by liver LDL receptors under normal conditions; thus, LDL receptor dysfunction leads to increases in plasma LDL concentrations [26, 27].

FA synthesis in the liver is regulated at the transcriptional level by insulin-activated sterol regulatory element binding protein 1c (SREBP-1c) and glucose-activated carbohydrate response element binding protein (ChREBP). In addition, the downstream regulatory targets of ChREBP, liver X receptor (LXR) and SREBP-1c include FAS, stearoyl-CoA desaturase-1 (SCD1) and glycerol-phosphate acyl transferase (GPAT) gene expression [28]. Positive regulation of these lipogenic enzymes directly leads to rapid increases in FA and TG synthesis and increases in the concentrations of monosaturated FAs (MUFAs) produced from saturated FAs (SFAs). To reduce FA/TG synthesis, hepatocyte farnesoid X receptor (FxR) activation inhibits SREBP and LXR activation and induces the expression of the FA mitochondrial oxidative factor peroxisome proliferator-activated receptor-alpha (PPARα) [29].

Impaired TG utilization may promote TG accumulation, and factors associated with this process include disruptions in liver FA β-oxidation, impairments in TG synthesis, and abnormal secretion of VLDL (Fig. 3) [30]. β-oxidation of FAs in mitochondria catalyses the decomposition of long-chain acyl-CoA into acetyl-CoA, and the regulation of transcription mainly involves PPAR, SREBP1 and peroxisome proliferator-activated receptor gamma coactivator-1α (PGC-1α). Posttranscriptional regulatory mechanisms are mainly involved in determining the expression of ACC, malonyl-CoA decarboxylase (MCD), carnitine palmitoyltransferase (CPT), and downstream genes as well as allosterically regulating β-oxidation [31]. Due to the very high intrahepatic and blood lipid levels in patients with NAFLD, the formation and accumulation of fat in the liver can increase both VLDL- Apolipoprotein B (ApoB) and VLDL-TG production rates (PRs) [32] (Fig. 3). TG synthesized by liver cells is combined with apolipoprotein B100 (ApoB100), MPT, and cholesterol to form VLDL, which is secreted into the blood by liver cells and transported to extrahepatic tissues [27]. When TG synthesized by hepatocytes is unable to form VLDL that enters the blood due to malnutrition, poisoning, essential FA deficiency, choline deficiency or protein deficiency, it accumulates in the hepatocyte cytoplasm to cause fatty liver [26, 27]. ApoB100 and microsomal TG transfer protein (MTP) mediate VLDL secretion in hepatocytes. Studies have revealed that pharmacological inhibition of MTP can be used to treat hyperlipidaemia in rats with monosodium L-glutamate-induced obesity, while increases in MPT gene or protein expression can be used to treat NAFLD [33–35]. Studies have also indicated that upregulating PPAR-α, carnitine palmitoyltransferase-1a (CPT-1a) and fatty TG lipase (ATGL) to enhance FA oxidation and lipolysis can improve NAFLD in micee [36, 37].

As shown in a study by Lutfi Abu-Elheiga [38], ACC2 deficiency prevents liver steatosis in mouse models of diet-induced obesity, fatty liver and diabetes. When the inhibition of CPT1 by ACC2 is reduced or abolished, the β-oxidation of liver FA is increased, and the synthesis of long-chain FAs and TG is significantly reduced to inhibit the development of fatty liver. Additionally, the decreases in acylglyceride levels in the livers of ACC2−/− mutant mice fed a high-fat, high-carbohydrate (HFHC) diet result in increases in p-AKT levels, which subsequently increase insulin sensitivity and enhance glucose uptake [38]. In an NAFLD mouse model, reductions in the expression of SREBP-1c in turn decrease the expression of downstream targeting enzymes such as ACC1, ATP citrate lyase (ACL) and FAS, which are important enzymes for DNL, thus decreasing lipid accumulation in the liver. Albert G. Linden et al. observed decreased levels of mRNA encoding enzymes related to glycolysis and lipogenesis in mice with liver-specific deletion of ChREBP (*L-Chrebp−/−* mice). These decreases resulted in reductions in DNL, TG synthesis and glycolysis [39]. These findings show that inhibition of key enzymes for hepatic lipid synthesis and key genes regulating the synthesis of these enzymes can reduce liver fat formation and accumulation.

This section mainly explains the processes of liver fat accumulation and fatty liver development resulting from imbalances between liver fat production and metabolism. The major mechanisms that cause liver fat accumulation include an increase in liver DNL, an increase in TG synthesis, a decrease in VLDL synthesis, and a decrease in β-oxidation of FAs. Importantly, the ER and mitochondria, which are important organelles for liver lipid synthesis and glycolipid metabolism, play vital roles in controlling liver fat production and metabolism. Since most of the enzymes involved in lipid synthesis and metabolism are localized to mitochondria and the ER, it is important to control the synthesis of these proteins (e.g., by controlling the genes that synthesize them).

### Activation of ER stress in patients with NAFLD

In response to increases in the ER flux of folded polypeptide chains, cells regulate the protein folding ability of the ER according to their physiological and environmental conditions, thereby ensuring high-fidelity synthesis of cell membrane structural proteins and the quality of the protein secretion mechanism. Many of the signalling pathways in cells that mediate this regulatory mechanism are incorporated into a process called the unfolded protein response (UPR) to maintain ER homeostasis [40]. When the UPR is not sufficient to maintain normal hepatocellular function, ER stress occurs in the cells, which can increase the disorder of ER-dependent liposome homeostasis, thus providing conditions for the development of steatosis [41].

**Fig. 4:**
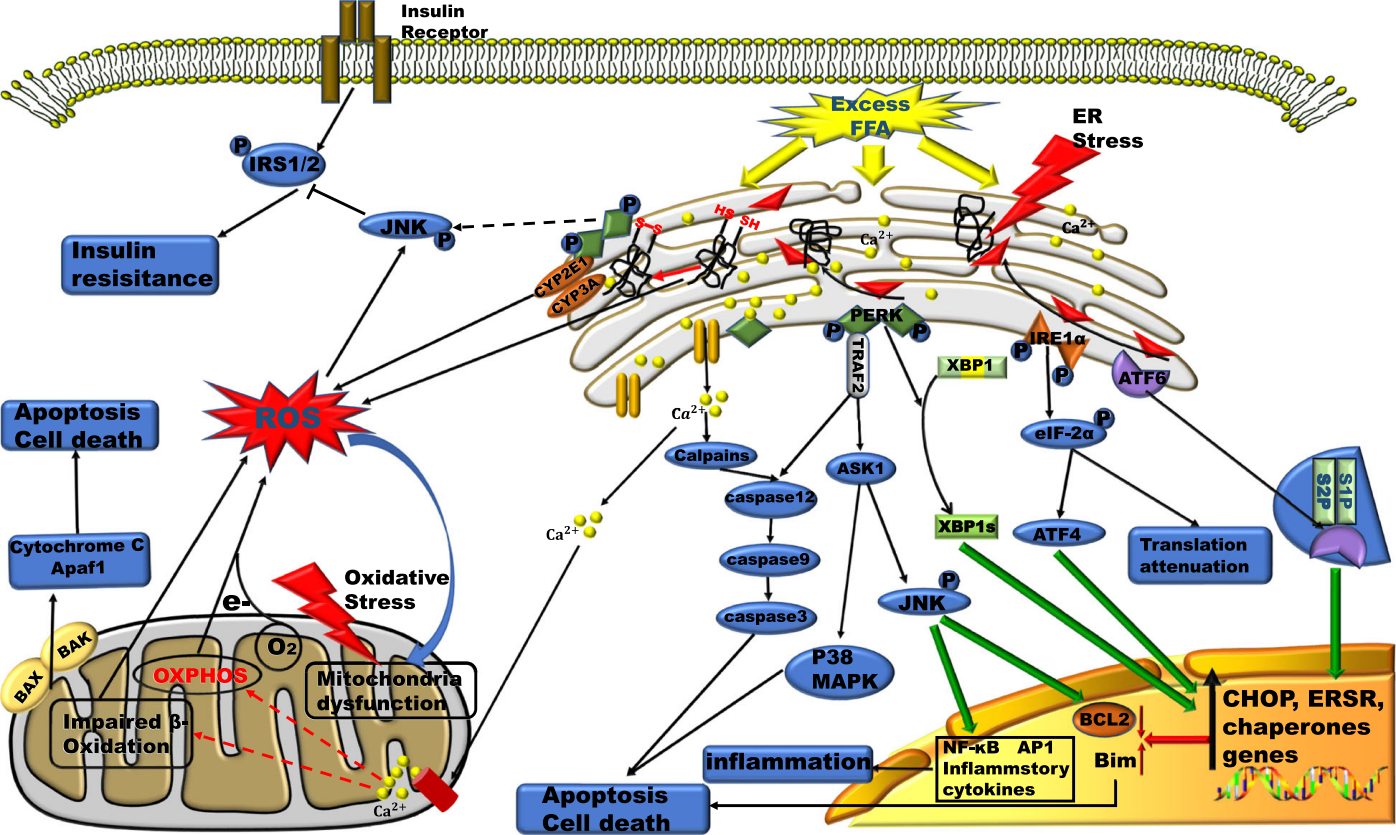
ERS and oxidative stress in patients with NAFLD and the generation and crosstalk of ROS signalling connecting these pathways. Excess accumulation of FFAs in hepatocytes induces ERS and oxidative stress, both of which are linked by ROS and Ca^2+^. a) When the UPR reaction fails to solve protein errors or unfolding, the ERS response may be delayed or insufficient, and hepatocyte apoptosis may be induced by ER and mitochondrial stress responses or other independent pathways. b) ROS and oxidative stress disrupt ER function, and Ca^2+^ release from the ER depends on ROS. Excess Ca^2+^ induces OMM permeabilization, which in turn increases mitochondrial ROS release. The increases in ROS levels will further increase intracellular Ca^2+^ levels. c) In this complex situation, ERS can induce apoptosis through a number of mechanisms, including mitochondrial damage leading to the production of apoptotic factors, cytochrome release, apoptotic body formation, and sequential activation of caspase-9 and caspase-3. IRE-1 recruits TRAF-2 to activate ASK-1 and JNK in a potential proapoptotic pathway that is further maintained by ROS or caspase-12. Activation of PERK and ATF6 (p90) leads to activation of ATF4 and ATF6, respectively (nuclear translocation of p50), and upregulation of CHOP expression, which inhibits Bcl-2 expression and promotes apoptosis

The chaperone protein glucose-regulated protein 78 (GRP78), also known as binding immunoglobulin protein (BiP), not only regulates the quality of folded proteins but also senses misfolded protein accumulation and then dissociates from protein kinase RNA-like ER kinase (PERK), inositol-requiring enzyme 1 (IRE1) and activating transcription factor 6 (ATF6). Therefore, the three core pathways of the UPR are activated. These UPR pathways ultimately act synergistically on the ER to increase the protein folding capacity, degrade misfolded proteins, and reduce the entry of new proteins [42]. Excessive accumulation of misfolded proteins promotes apoptosis through a process that may be mediated by Ca2+ disorder, excess ROS production, and oxidative stress [43] (Fig. 4). Compared to normal liver tissues, NAFLD tissues have been detected to have higher levels of C/EBP homologous protein (CHOP), cleaved ATF6 and X-box binding protein 1 (XBP1, an mRNA that is spliced by IRE1), indicating that the UPR and ERS are activated in subjects with NAFLD [44].

BiP is highly concentrated in the ER lumen and preferentially binds to unfolded proteins; thus, it can be depolymerized by IRE1, PERK and ATF6 only when the amount of unfolded protein is large. However, a small amount of protein accumulation may cause a UPR reaction. In fact, in addition to being activated by unfolded proteins, the UPR can also be activated by lipid bilayer stress (LBS) [45].

Despite the lack of a sensing domain for unfolded proteins in the ER lumen, IRE1 and PERK in mammalian cells can detect perturbations in the lipid composition of the ER membrane. In vitro experiments have shown that increased acyl chain saturation of the ER transmembrane domain (TD) activates PERK and IRE1. LBS can enhance dimerization via the TD of the ER membrane such that the TD is incorporated into an oligomer, which promotes the activation of IRE1 and PERK to some extent [46]. Upon sensing of LBS, IRE1α dimerisation or the formation of higher oligomers from monomers is induced, followed by trans-autophosphorylation. Subsequently, the IRE1α/XBP1, PERK/p-eIF2α and ATF4 UPR pathways are activated, and the final effect is identical to that caused by unfolded proteins [47]. LBS promotes the accumulation of dihydrosphingosine (DHS) or dihydroceramide (DHC), which facilitates the bending of the ER lipid bilayer membrane, followed by activation of IRE1 and ATF6 [47, 48] (Fig. 4). Fu S et al. found that although ER-related protein synthesis is reduced in obese fatty liver, chronic ERS exists in the livers of mice. It can be speculated that ERS in fatty liver may be caused not only by protein overload but also by impaired folding ability [49].

Accumulation of several hepatic lipids has been observed in the livers of mice treated with tunicamycin (a classic ERS inducer), which can induce phosphorylation of PERK, increase the expression levels of Chop and Grp78 and inhibit the expression of ApoB100 [50]. As shown in a study by Shen C et al., palmitic acid is likely to cause hepatic lipotoxicity by activating the TLR4-IRE1α pathway. Specific deletion of TLR4 in the liver has a protective effect in mice with diet-induced NAFLD [51]. Another study has indicated that after high-fat diet consumption, wild-type mice with overexpression of a growth arrest and DNA damage gene (GADD34, which dephosphorylates eIF2α) show reduced liver TG levels [52]. The PERK/p-eIF2α/ATF4 pathway induces the formation of NAFLD by initiating a lipogenesis-related process. Zeng L et al. have shown that ATF6 inhibits the transcription of SREBP-2 by forming a complex with SREBP-2, which in turn inhibited fat synthesis in HepG2 and HEK293 cells [53]. Induction of the expression of ATF6 in C3H10T1/2 cells has been found to significantly decrease the levels of lipid-generating transcription factors such as PPAR-γ and to inhibit C3H10T1/2 cells from differentiating into mature lipid cells [54]. These findings further demonstrate that ATF6 is involved in the expression of lipid synthesis genes and thus modulates lipid synthesis.

During ERS, accumulation of excess misfolded proteins due to the limited compensatory capacity of the ER to clear misfolded proteins can activate autophagy [55]. Activation of the PERK-eIF2α-ATF4 pathway and the IRE1-JNK pathway promotes autophagy, which ensures, to some extent, normal energy metabolism and immune responses in cells, ultimately promoting cell survival [50]. Autophagy reduces the level of ERS mainly by removing the ER membrane portion containing the UPR sensor and clearing abnormal proteins in the ER [55]. C-X-C motif chemokine receptor 3 (CXCR3) is closely associated with autophagosome/lysosome damage and ERS in subjects with NASH, as evidenced by LC3-II and p62/SQSTM1 accumulation and increased levels of GRP78, p-PERK and p-eIF2α. CXCR3 antagonists inhibit MCD-induced steatosis and hepatocyte damage in AML-12 hepatocytes. In vivo experiments in mice have shown that CXCR3 antagonists block CXCR3 activity and reverse NASH [56].

In summary, the UPR is caused by protein overload and impaired folding ability. When the UPR is insufficient to maintain normal liver cell function, ERS occurs in cells. The activation and maintenance of liver ERS occur mainly through the activation of signalling pathways such as the PERK/XBP1, IRE1α/eIF-2α/ATF4, and ATF6 pathways, which promote liver lipid accumulation, liver cell inflammation and apoptosis. Targeting these proteins or genes involved in ERS activation has been shown to ameliorate NAFLD development.

### Production, communication and impact of ROS in the liver mitochondria, ER and MAMs

The ER produces ROS through two main pathways (Fig. 4). First, during the process of disulfide bond formation at thiol groups, ER oxidoreductin-1 (ERO1) and protein disulfide isomerase-1 (PDI1) cooperate to transfer electrons from the thiol group of the substrate protein to molecular oxygen while producing ROS as a byproduct [57]. Second, this “electron leakage”, which results from the low efficiency or degree of coupling of electron transfer from NADPH to p450 in the microsomal monooxygenase (MMO) system, plays a significant role in ROS generation, especially in hepatocytes [58]. High levels of ROS are produced in the ER, and ROS mainly act on the Ca2+ channel receptor on the MAM, resulting in extensive release of ER Ca2+ into the mitochondria. High concentrations of Ca2+ due to new steroids promote mitochondrial oxidative disorders, which lead to oxidative stress [58]. ROS produced in the ER can indirectly affect mitochondrial oxidation, regardless of whether mitochondria are affected through direct action, because of ROS transport. Therefore, we suspect that ROS can directly interact with MAMs and disrupt their respective functions. In the mitochondria, the ROS-producing system generally includes the mitochondrial respiratory chain or oxidative phosphorylation, the hepatic cytochrome P450 system, the protein autoxidation system, the NADPH oxidase complex and the xanthine oxidase system (Fig. 4) [59]. Interestingly, the production of ROS by certain mitochondria triggers the production and release of ROS from adjacent mitochondria. The mutual crosstalk among these ROS activates ROS-mediated signal transmission networks in different locations that are not subject to spatial constraints, and these pathways interconnect and further enhance the activity of other ROS signalling pathways [60].

Imbalances between FFA synthesis and degradation are regulated by shifts in metabolism, including enhanced β-oxidation of mitochondrial FAs, induction of TCA cycling, and stimulation of oxidative phosphorylation. All of these processes produce ROS, thus accelerating the accumulation of ROS and promoting cellular inflammation and apoptosis by activating pro-inflammatory signalling pathways (such as the MAPK, NF-κB and JAK-STAT pathways) [61]. In addition, interference with TCA cycle function may lead to mitochondrial oxidative and respiratory dysfunction and increase liver ROS levels, oxidative stress, and activation of the liver inflammatory pathway, all of which contribute to the progression of NAFLD (Fig. 4). During IR, the hepatic TCA cycle increases electron deposition into a relatively inefficient oxidative respiratory chain that readily generates ROS and provides a mitochondria-derived substrate for elevated gluconeogenesis; these effects work together to produce mitochondrial dysfunction [62].

Under normal physiological conditions, the production and elimination of ROS and processes of cellular damage and self-repair are in dynamic equilibrium. Autophagic, apoptotic and fibrogenic signalling pathways are activated when high levels of ROS are produced and antioxidant levels are not sufficient to eliminate them. Moreover, the integrity of cell membranes and the normal functions of proteins and DNA are all disturbed and destroyed by the effects of ROS [63]. The antioxidant defence mechanism eliminates ROS by producing antioxidants such as glutathione and superoxide dismutase [64].

Overall, lipid accumulation exacerbates ROS production in liver mitochondria and the ER. ROS can engage in crosstalk and communication between the ER and mitochondria through MAMs. Interestingly, production of large amounts of ROS during ERS disrupts Ca2+ homeostasis and the ROS balance between mitochondria and the ER or even the cytoplasm overall, further disrupting mitochondrial function and leading to increased mitochondrial ROS production. The production of large amounts of ROS is the main cause of oxidative stress in liver cells. When the liver’s own antioxidant capacity is insufficient to clear ROS or when oxidative stress persists, NAFLD-related apoptosis, autophagy, inflammation and fibrosis are activated, accelerating the progression of NAFLD. Oxidative stress is another important factor in the development of fatty liver.

## ER-mitochondria coupling

### MAMs

Direct physical interaction between the ER and mitochondria is limited in cells and essentially does not involve membrane fusion. The interaction and communication are mediated by membrane proteins, enzymes involved in lipid synthesis, and some cellular signalling molecules that are also reversibly linked to membrane regions corresponding to mitochondria [65].

**Fig. 5:**
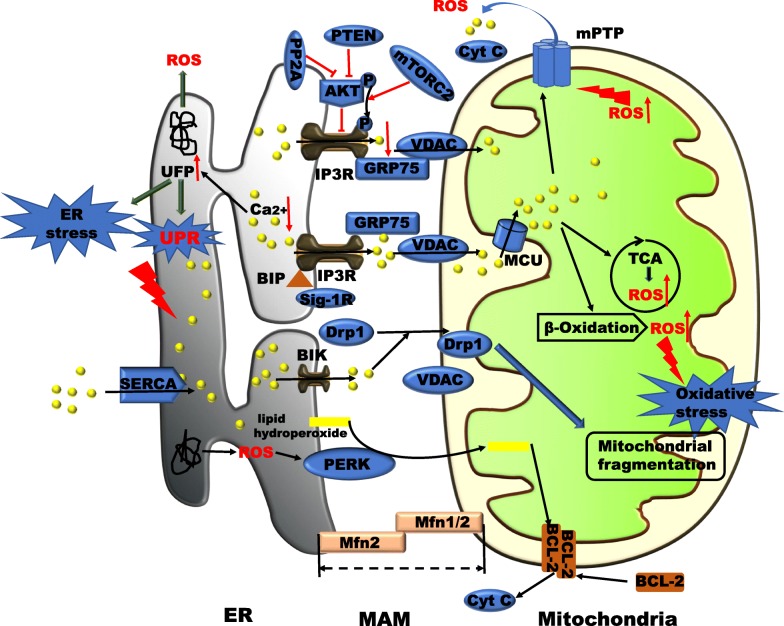
Representation of intracellular Ca^2+^ dynamics and MAM proteins involved in ER–mitochondria Ca^2+^ crosstalk in patients with NAFLD. A range of proteins (e.g., PML, Akt, GRP-75, SIG-1R, Mfn1/2, and BiP) located in MAMs regulate Ca^2+^ release from the ER and efficient mitochondrial Ca^2+^ uptake, resulting in different consequences. FFAs stimulate the ER in patients with NAFLD, thereby promoting the transport of Ca^2+^ ions from the ER to MAMs or the cytoplasm by IP3R. The formation of locally high Ca^2+^ concentration microdomains drives Ca^2+^ into the mitochondrial matrix through VDAC and MCU, and ER Ca^2+^ transfer is enhanced by GRP75. Increases in mitochondrial Ca^2+^ concentrations promote the production of NADH by enzymes in the TCA cycle and increase ATP synthesis and ROS production. Sustained increases in Ca^2+^ concentrations promote mPTP opening followed by the release of cytochrome c (cyt c) and the induction of apoptosis. In addition, PERK may promote the rapid transfer of ROS signals to MAMs in the form of lipid hydroperoxides. As a result, mitochondrial phospholipids are oxidized, and cyt c is released from the mitochondrial pool in close contact with the ER. Interestingly, after transmission of the apoptotic signal, the BH3 family member Bik induces Ca^2+^ release from the ER, which in turn induces the recruitment of Drp1 to the mitochondria. Reductions in ER Ca^2+^ ion concentrations increase the production of unfolded proteins or misfolded proteins, leading to ERS and ROS production. ROS or their signals cause further disturbances in Ca^2+^ homeostasis, creating a vicious positive feedback cycle

Long-chain acyl-CoA synthetase 4 (ACSL4), encoded by a gene of the same name, mediates the attachment of lipid FAs to CoA and participates in the synthesis of TAG. Notably, this enzyme is considered one of the most reliable MAM marker proteins [66]. Previous reports have demonstrated that ACSL4 expression is elevated in the livers of NAFLD patients or animals [67, 68]. Amar B. Singh et al. compared high-fat diet (HFD)-fed ACSL4 knockdown (KD) mice with HFD-fed normal wild-type mice and found that plasma VLDL-TG, glucose metabolism and liver phospholipid synthesis require ACSL4 expression in the liver [69]. Serum VLDL-TG in ACSL4 KD mice was significantly reduced, while liver and plasma cholesterol levels were not significantly different between the groups, indicating that ACSL4 may direct PUFAs into the hepatocyte TG synthesis pathway [69]. Voltage-dependent anion channel (VDACs) in the outer mitochondrial membrane (OMM) are physically linked to the ER Ca2+ release channel inositol 1,4,5-triphosphate receptor (IP3R) by GRP75 (a portion of which appears to be in the cytosol at the MAMs) and serve as channels for signalling molecules, including Ca2+, apoptotic signals, and metabolites. Recombinant expression of the ligand binding domain of IP3R on the surface of the ER or mitochondria increases the accumulation of Ca2+ in the mitochondria [70] (Fig. 5). Mitofusin 1/2 (Mfn1/2) has recently been shown to exert a negative regulatory effect on the distance between the ER and mitochondria. Mfn1/2 interaction increases the distance between the organelles and prevents excessive contact. KD of Mfn2 in mouse embryonic fibroblasts (MEFs) decreases the distance between the ER and mitochondria and increases Ca2+ transfer [71–73]. Interestingly, other studies have reported that Mfn2 acts as a link between ER-bound mitochondria and the ER and that Mfn2 ablation increases the distance between ER and mitochondria and reduces mitochondrial Ca2+ ion absorption in MEFs and HeLa cells [74, 75].

Other possible elements that link the ER and mitochondria include ER chaperones, particularly Ca2 + −binding chaperones (calnexin, calreticulin and BiP); a member of the dynamin family of GTPases called dynamin-related protein (Drp1, also known as DVLP, DLP1 or dymple); tumour autocrine motility factor receptor; and the apoptosis regulatory proteins phosphofurin acidic cluster sorting protein 2 (PACS2) and B-cell-receptor-associated protein 31 (BAP31) [76, 77]. Findings of increased levels of the 66-kDa isoform of the growth factor adaptor Shc protein (p66Shc) in MAMs and increased ROS production in crude mitochondria (containing MAMs) have revealed crucial roles for p66Shc in ROS production, transfer and accumulation in MAMs [78]. The ultrastructure of MAMs is closely related to some signalling pathways involved in metabolism, oxidative stress, ERS, inflammation, and apoptosis in cells. The plasticity of MAM structures is controlled by tethering complexes and crucial MAM proteins, such as PACS-2, Mfn2, Ca2+ channel transient receptor potential protein 2 (TRPP2) and some chaperones of the ER and mitochondria [79].

Galmes R et al. have shown that the mammalian oxysterol-binding protein (OSBP)-related proteins ORP5 and ORP8 are localized to MAMs. They target MAMs and interact with mitochondrial PTPIP51 depending on a functional lipid binding/transfer (ORD) domain. In addition, the normal morphology and function of mitochondria are disrupted in ORP5/ORP8-silenced cells [80]. György Csordás et al. used electron tomography (ET) to analyse isolated rat liver mitochondria, and the results showed that narrow particles connected OMMs to putative ER vesicles [81]. The length of the tethers connecting OMMs and the smooth ER in situ was 9–16 nm, and the length of the tethers connecting OMMs and the rough ER in situ was 19–30 nm [81]. In addition, the researchers compared MAMs between IP3R triple knockout (IP3R-TKO) DT40 cells and wild-type cells and found that IP3R-TKO DT40 cells had ER–mitochondria associations similar to those of wild-type cells. These results indicate that there are IP3R-independent physical connections between the ER and mitochondria [81].

MAMs are functional domains between two organelles that are involved in Ca2+ exchange through the VDAC-1/Grp75/IP3R-1 complex and regulate energy metabolism. In addition, many signalling proteins involved in physiological responses, such as metabolism, oxidative stress, ERS, inflammation and apoptosis, are located in MAMs, which together form the complex structures of MAMs [79]. The special structures of MAMs determine their involvement in mitochondrial and ER-related functions. Therefore, restoration of the structures and functions of MAMs may inhibit or reverse the occurrence and development of NAFLD.

### Effects of glucose and lipid metabolism on the structures and functions of MAMs

As mentioned above, the mitochondria and ER membranes contain most of the enzymes required for TG synthesis, and these enzymes are also abundantly enriched in MAMs, which play a key role in processing during lipid synthesis. The synthesis and processing of phosphatidylcholine (PC), phosphatidyl ethanolamine (PE) and cholesterol are also accomplished in MAMs [23, 82, 83]. Furthermore, MAM integrity is required for insulin signalling and glucose-sensing systems, and certain regulatory factors involved in glucose regulation that are affected by MAM integrity and metabolic homeostasis are also located in this region [82]. PA is converted to phosphatidylserine (PS) in the ER by phosphatidylserine synthetases 1 and 2 (PSS1 and PSS2, respectively). PS is transported to the mitochondria by MAMs, where phosphatidylserine decarboxylase (PSD) decarboxylates PS to form PE. The PE synthesized in the mitochondria is transported back to the ER by MAMs, and PC is synthesized by phosphatidylethanolamine N-methyltransferase (PEMT) [83]. Biochemical separation of MAMs and purification of the ER have been used to detect the enzymatic activity of PSS1 and PSS2, and the activity of both enzymes is closely related to MAMs rather than to purified ER [84]. MAMs are the exclusive sites at which PEMT2 exerts its activity and catalyses the final synthesis of PC. Upon inhibition of the hepatic expression of PEMT in a mouse model, the severity of liver damage has been found to increase proportionally with the extent of inhibition of the protein levels and activity of PEMT [85]. According to Gonçalves, exercise reduces the PC/PE ratio in rats and improves NAFLD symptoms to some extent [86]. Most of the enzymes involved in the synthesis and regulation of PC/PE are localized to MAMs, so maintaining the integrity and normal function of MAMs is conducive to improving NAFLD. Fu S et al. have shown through lipid metabolomics analysis in mice that upregulation of the key genes phosphate cytidylyltransferase 1, choline, alpha (Pcyt1a) and Pemt leads to increases in PC synthesis in the ER in fatty liver and to increases in PE conversion to PC or the PC/PE ratio [49]. In addition to phospholipid metabolism, steroidogenesis also has a certain effect on the development of NAFLD. Francisco Caballero et al. found that the mRNA levels of steroidogenic acute regulatory protein (StAR) (which can transport the substrate cholesterol into the mitochondria for steroidogenesis) in steatosis and NASH patients are 7- and 15-fold higher, respectively, than those in controls [87].. StAR interacts with VDAC2 in MAMs prior to translocation into the mitochondrial matrix, suggesting that MAMs are the sites of mitochondrial steroidogenesis [88]. These results indicate that increases in the levels of steroids (mainly free cholesterol) in MAMs may promote the development of NAFLD.

The primary factors regulating MAM integrity are glucose levels, and the effects of glucose levels on MAMs have been replicated both in vivo and in vitro. Theurey et al. compared the numbers of mitochondria–ER contact sites (MERCs) in mice that were fasted to the postprandial numbers and found that elevated glucose levels during this process increased the numbers of MERCs [89]. During screening of related metabolic signalling molecules, high glucose levels were found to disrupt the integrity and function of MAMs by activating the pentose phosphate-2A (PP2A) pathway, thereby inducing autophagy, division and respiratory damage in the mitochondria [88, 89]. MAM integrity has been reported to determine the activity of the insulin signalling pathway and the hepatic glucose-sensing system, and induction of MAMs effectively prevents palmitate-induced alterations in insulin signalling in HuH7 cells [89]. IR and dysregulated glucose and lipid metabolism are recognized as important risk factors for NAFLD. On the one hand, IR decreases glucose uptake by cells and leads to increases in circulating FFA levels. FFAs are more likely to accumulate in the liver under these conditions, leading to increased hepatic fat accumulation and fatty liver. On the other hand, IR leads to increased degradation of fat in white adipose tissue (WAT), again increasing FFA levels [90]. Excessive FFA levels in hepatocytes stimulate the ER and mitochondria to initiate ERS, oxidative stress, and subsequent inflammation and apoptosis and thus further aggravate the progression of NAFL to NASH [91].

As bridges between the ER and mitochondria with regard to structural and functional communication, MAMs are required for phospholipid metabolism, steroid metabolism, glucose metabolism, and FA metabolism in the liver. The integrity of MAMs determines the normal transmission of PC/PE ratios and glucose signals, and glucose levels regulate MAM integrity. The transmission of abnormal glucose signals leads to IR, which further causes ERS and oxidative stress and ultimately promotes the occurrence and development of NAFLD. Therefore, normal MAMs can regulate PC/PE ratios and ensure normal glucose signals, which are often used as targets for ameliorating NAFLD development.

### Regulation of Ca^2+^ ion homeostasis in MAMs

MAMs are involved in the transport of Ca^2+^ from the ER to the mitochondria. This interaction is important for the rapid uptake of Ca^2+^ by the mitochondria through VDACs, which are located in the OMM. The transport of Ca^2+^ between the ER and mitochondria by MAMs relies on the regulatory protein IP3R and the chaperone protein GRP75; these proteins are important components of MAMs. Ca^2+^ transport is mainly affected by the ER Ca^2+^ concentration and relies on the mitochondrial Ca^2+^ unidirectional transporter (MCU) for mitochondrial Ca^2+^ uptake to produce the electrochemical gradient [83, 92]. Agonists or first messengers bind to receptors on the cell membrane surface to produce the second messenger IP3, which binds to IP3R and promotes the release of Ca^2+^ from the ER into the MAMs and cytosol [92]. IP3R, VDAC and GRP75 form very abundant and large voltage-gated pores in MAMs that mediate Ca^2+^ transport (Fig. 5).

Arruda et al. have shown that liver MAM levels and the expression of Ca^2+^ flux proteins associated with MAMs are increased in obese mice; increased accumulation of Ca^2+^ in mitochondria leads to mitochondrial dysfunction, increased ROS production, cellular stress, impaired insulin action in the liver, and abnormal glucose metabolism (e.g., liver steatosis and glucose intolerance). Downregulation of IP3R1 and PACS-2 can ameliorate these abnormal pathological conditions in obese mice [93]. However, Tubbs et al. suggested that increasing the integrity of MAMs could help restore mitochondrial function and improve IR in mice [94]. These findings seem to contradict each other. However, we can speculate that if the pathology of the obese/fatty liver model obtained by Arruda et al. involves excessive increases in MAMs, the model established by Tubbs et al. does not exhibit reduced MAM integrity. Although these two conditions develop towards different extremes, they have certain similarities (mitochondrial dysfunction and IR). These two conditions are related to many factors, including an individual’s metabolic state, age and stress and even the different experimental conditions.

Several proteins involved in insulin signalling that are located on MAMs, including protein kinase Akt (also called PKB), PP2A, mammalian target of rapamycin complex 2 (mTORC2), and phosphatase and tensin homologue (PTEN), also regulate the transport of Ca^2+^ by IP3R [95] (Fig. 5). IP3R is phosphorylated by Akt to inactivate and shut down channels, thereby reducing Ca^2+^ release. Dephosphorylation of Akt by PP2A eliminates the inhibitory effect of Akt on IP3R, and Akt-mediated phosphorylation and inhibition of IP3R are restored by PTEN, thus restoring Ca^2+^ flow between the ER and mitochondria [95, 96]. Furthermore, mTORC2 phosphorylates and activates Akt, which promotes the phosphorylation of IP3R and reduces Ca2+ release [97, 98].

Previous reports have shown that the activity of sarco−/endoplasmic reticulum Ca^2+^-ATPase (SERCA) on the ER in liver L02 cells is inhibited during the development of NAFLD, resulting in decreased Ca^2+^ entry into the ER and increased Ca^2+^ transport from the ER to the mitochondria. When palmitic acid is used to reduce the activity of SERCA in vitro, severe imbalances in Ca^2+^ homeostasis and subsequent ERS are observed [99]. After the recovery of SERCA activity (restoration of Ca^2+^ homeostasis), the levels of the ERS-related markers GRP78, SREBP, p-PERK, p-eIF2α, and CHOP are reduced, inhibiting the progression of NAFLD in L02 and BEL-7402 hepatoma cells [99, 100]. By modulating the oxidative modification of SERCA2b, Cisd2 can induce Ca^2+^ pumping activity, thereby increasing ER Ca^2+^ uptake, maintaining Ca^2+^ homeostasis, inhibiting ERS, and protecting against or ameliorating NAFLD [101]. The mechanism by which Ca^2+^ activates the UPR mainly involves two factors. First, protein folding is a Ca^2+^-dependent process, and the production and accumulation of misfolded proteins are closely related to the consumption of Ca^2+^ stores in the ER lumen. Second, excess Ca^2+^ depletion results in the binding of GRP78 to unfolded proteins and the subsequent detachment of GRP78 from IRE1, PERK and ATF6 [102]. Excess or chronic reductions in ER Ca^2+^ concentrations alter the functions of chaperone proteins, which in turn induces ERS and affects the initiation and development of NAFLD.

Intriguingly, in addition to exerting effects on the UPR, ROS produced by ERO1α also stimulate IP3R-mediated Ca^2+^ release [57]. The subsequent consumption of ER Ca^2+^ stores further increases the production and accumulation of unfolded proteins and exacerbates ROS production [103]. Therefore, a vicious positive feedback loop forms, which further expands the effects of ROS-activated IP3R release from ER Ca^2+^ stores. On the other hand, as IP3R is continuously activated, large amounts of stored ER Ca^2+^ are released into the mitochondria and cytoplasm, eventually leading to mitochondrial Ca^2+^ overload and subsequent ROS production and mitochondrial dysfunction. Deletion of PERK disrupts the integrity of MAMs, reduces ROS entry into the mitochondria and ER, and inhibits ROS-induced mitochondrial dysfunction and apoptosis [104, 105]. PERK is enriched in MAMs, and along with ER fragmentation and ER Ca^2+^ handling disturbance, protection of mitochondria in PERK^−/−^ MEFs from ROS-mediated apoptosis occurs (Fig. 5). According to studies by Verfaillie et al. [105], PERK increases the exchange of ROS between the ER and mitochondria by maintaining the levels of the proapoptotic protein CHOP, leading to apoptosis in MEFs. In contrast, studies by Muñoz et al. have revealed that KD of PERK in MFN2-deficient MEFs reverses mitochondrial network fragmentation and restores mitochondrial Ca^2+^ levels. Additionally, chronic activation of the PERK pathway has been detected in *Mfn2*-knockout (KO) MEFs, livers and skeletal muscles [106]. Fu S et al. found that the activity of SERCA in liver-derived microsomes is significantly inhibited by the addition of exogenous PC or PEMT in mice. Therefore, it is likely that increases in PC/PE in the liver ER may disturb the Ca^2+^ homeostasis of the ER and cytoplasm, leading to protein misfolding and ERS. The researchers also concluded that a vicious fatogenesis-ERS-fatogenesis cycle led to NAFLD in mice and that restoring PC/PE balance and Ca^2+^ homeostasis may improve ERS-related NAFLD [49].

The ER acts as a cell’s Ca^2+^ ion reservoir, maintaining a steady state of Ca^2+^ ions in the cytoplasm and mitochondria and in the ER itself. MAMs mediate Ca^2+^ transport and regulate related proteins and their functions, fully demonstrating the important role of MAMs in maintaining mitochondrial Ca^2+^ ion homeostasis. MAM-mediated Ca^2+^ ion conduction can not only affect cell survival and energy metabolism but also participate in related signal transduction processes, such as those associated with IR. Many studies have shown that in NAFLD individuals, the destruction of hepatocyte Ca^2+^ homeostasis is mainly due to abnormalities in MAMs, such as abnormal expression of certain proteins. Destruction of Ca^2+^ homeostasis in MAMs is related to IR, ROS production, ERS, and mitochondrial dysfunction. These factors are important causes for the development of NAFLD.

### NAFLD-specific consequences of MAM disruption

ERS has an important relationship with the occurrence of IR, particularly in peripheral tissues such as the liver and adipose tissues in mice and in corresponding cells. Secreted insulin activates the tyrosine kinase cascade, and its substrate (IRS-1) also undergoes tyrosine phosphorylation, activating the downstream PI3K and MAPK pathways [107]. Disruption of interorganelle Ca^2+^ transfer is associated with ERS, mitochondrial dysfunction, lipid accumulation, and activation of JNK, protein kinase C ε and IR in the livers of CypD-KO mice [108]. MAMs act as main channels for Ca^2+^ exchange between the ER and mitochondria, maintaining the homeostasis of both intramembrane and cytoplasmic Ca^2+^. Therefore, the integrity and spatial structures of MAMs exert critical effects on liver insulin signalling [108, 109].

Ozcan et al. used thapsigargin to establish a hepatocyte ERS model and showed that ERS impedes the insulin receptor signalling pathway. Under these conditions, JNK-dependent serine phosphorylation of IRS-1 is increased, and tyrosine phosphorylation of IRS-1 is decreased. The inhibitory effects of ERS are significantly attenuated when the JNK signalling pathway is inhibited. ERS causes IR by promoting serine phosphorylation of IRS-1, blocking the insulin receptor signalling pathway and impeding the absorption and utilization of glucose in the human body [110]. As shown in a study by Guo Q et al., the novel adipokine progranulin induces IR by promoting autophagy caused by oxidative stress and ERS in 3 T3-L1 adipocytes and C57BL/6 J mice [111]. IR has consistently been reported to serve as a risk factor for NAFLD because it causes dysfunctional glycolipid metabolism in the liver, resulting in hepatic fat accumulation [112].

Lipotoxicity-induced Ca^2+^ disturbances activate the UPR during hyperlipidaemia-induced fatty liver disease in mice [113]. Jie Ning et al. used C57BL/6 mice with HFD-induced IR as a research model and found that the IRE1/XBP-1 pathway induces the expression of the lipid synthesis genes SREBP-1c and FAS in primary hepatocytes, thereby inducing hepatic lipid metabolism dysfunction. Furthermore, long-term exposure to insulin activates the IRE1α-XBP1 pathway through a mechanism that depends on the synthesis of mammalian target of rapamycin (mTOR)-dependent proteins [114]. The mTOR protein plays a crucial role in maintaining Ca^2+^ homeostasis in MAMs. Therefore, we speculate that mTOR may reduce ERS by maintaining Ca^2+^ homeostasis, resulting in suppression of the UPR and the IRE1α-XBP1 signalling pathway. However, under long-term chronic fasting conditions, hepatocyte-specific elimination of IRE1α destroys FA β-oxidation and ketone production in the liver, leading to hepatic steatosis; liver-specific recovery of downstream XBP1s reverses this deficiency in IRE1α^−/−^ mice [115]. In the course of the development of fatty lesions in the liver, ERS causes abnormal liver lipid metabolism by interfering with the homeostasis of Ca^2+^ and ROS. However, this effect is mutual; excess lipid deposition induces ERS. As described above, lipid accumulation induces ERS through the FFA-Ca^2+^ homeostasis-UPR axis. The basis for all of these consequences is that MAMs act as bridges to accelerate crosstalk between the ER and mitochondria.

Oxidative stress mediates inflammation, autophagy, and apoptosis in liver cells, and these processes can exacerbate the progression of NAFLD. The “three-hit” hypothesis states that under oxidative stress, ROS activity in the liver is increased, large numbers of inflammatory cytokines are released, and inflammation develops in liver cells to form NASH. Long-term persistence of NASH in the body gradually increases liver inflammation and cell necrosis, and NASH eventually develops into fatty liver, fibrosis and cirrhosis [116]. A consequence of ERS is the accumulation of ROS, which promotes a state of oxidative stress. Through activation of the nuclear factor E2-related factor 2 (Nrf2) and ATF4 transcription factors, PERK signalling coordinates the convergence of ERS with oxidative stress signalling [117]. After the PERK or Nrf2 gene is silenced in ρ zero cells (obtained from wild-type and Nrf2−/− murine embryo fibroblasts), reduced glutathione levels decrease, ROS levels significantly increase, and the numbers of apoptotic cells increase. Thus, in response to oxidative stress, the sensitivity of cells to the ERS-induced pathological response increases, and the PERK/Nrf2 pathway plays a role in regulating oxidation [118]. According to Sharma et al., pharmacological activation of Nrf2 reverses IR by inhibiting ERS, inflammation and oxidative stress; inhibits hepatic steatosis; and alleviates NASH and liver fibrosis in obese and insulin-resistant mice [119]. Therefore, oxidative stress is not only a factor predisposing an organism to ERS but also an important contributor to ERS-induced cellular damage.

Hepatocyte apoptosis has consistently been shown to promote the occurrence and development of NASH [120]. In liver tissues from patients with NASH, the UPR-related PERK/ATF4/CHOP signalling pathway is activated, and blockade of this pathway partially inhibits hepatocyte apoptosis [121]. Previous ERS research has focused on the UPR, but non-UPR pathways have gradually attracted the attention of researchers. Non-UPR pathways include the GSK-3 pathway, the PI3K/Akt pathway, etc., which induce the development of NAFLD through lipotoxicity-induced ER-related apoptosis [107].

Hernández-Alvarez MI et al. induced NAFLD by MCD diet feeding and Mfn2 KD and found that Mfn2 expression was significantly decreased in the livers of mice with MCD-induced NAFLD. Lack of Mfn2 reduces the transfer of PS from the ER to the mitochondria, and the reduced expression of PSS1 and PSS2 due to compensatory inhibition inhibits PS synthesis, ultimately leading to phospholipid metabolism impairment. Damage to phospholipid metabolism activates the expression of the UPR-related proteins PERK, p-IRE1, p-eIFα, and ATF6. In addition, liver Mfn2 ablation causes MAM remodelling, which leads to fat accumulation and IR in the livers of obese and diabetic mice [122].

Transfer of PS also occurs between the ER and mitochondria, and the PE formed by PS on the mitochondria maintains mitochondrial morphology and respiratory function [80, 123]. Such findings illustrate the important regulatory role of Mfn2 in NAFLD. Interestingly, Galmes R et al. also found that ORP5 and ORP8 specifically bind to PS and transfer it from the ER to the plasma membrane and mitochondria in cells [80, 124]. Therefore, we can speculate that ORP5 and ORP8 also affect NAFLD by regulating MAMs.

The unique structures and functions of MAMs mediate their roles as bridges in regulating mitochondria and the ER. The cellular signals or biochemical reactions related to MAMs mainly include IR, ERS, glucose and lipid metabolism, oxidative stress, and apoptosis. These processes may be involved in the occurrence and development of NAFLD. Disruption of MAM integrity is mainly reflected by changes in spatial distance and the levels of related proteins. The accumulation of lipids in the livers of NAFLD patients with damaged MAM integrity leads to Ca^2+^ ion disturbance, which accelerates ERS and oxidative stress, further leading to IR, apoptosis, and inflammation, which accelerate the progression of NAFLD. In addition, crosstalk among these processes through MAMs exacerbates liver fat accumulation, inflammation and apoptosis. MAMs may be the main amplifiers of these consequences.

## Drug treatment for NAFLD

Current clinical treatments for NAFLD mainly aim to promote weight loss and proper exercise and include the administration of insulin sensitizers and antioxidants. However, many interconnected and complex pathways are involved in the onset of NAFLD, suggesting that monotherapy is unlikely to be an effective treatment strategy. Therefore, a joint intervention targeting multiple pathways is ultimately needed [116]. Some of the drugs or compounds described below that are associated with MAMs may be relevant to the treatment of NAFLD (Table 1).

**Table 1:** Drugs or compounds identified to have therapeutic effects on NAFLD and their mechanisms related to MAMs

Drug or compound	Animals	Mechanism(s)	Reference(s)
Rosiglitazone	*ob/ob* and diet-induced insulin-resistant mice, *CypD*-KO mice	Increased MAM numbers in primary hepatocytes with overexpression of CypD, VDAC1 and PACS2, enhanced insulin signalling and action.	[94]
Metformin	*ob/ob* and diet-induced insulin-resistant mice, CypD-KO mice	Improved ER-mitochondria interactions.	[94, 125]
Forskolin	HuH7 cells treated with glucose	Prevention of high glucose-mediated reductions in MAM integrity	[89]
Sulforaphane	*ob/ob* mice and HFHSD mice, primary mouse hepatocytes treated with palmitate in vitro	Improved glucose tolerance, MAM protein content and ER–mitochondria interactions; decreased levels of the ERS markers CHOP and Grp78	[125, 126]
Hepatic stimulator substance (HSS)	Mice fed a methionine and choline-deficient diet	Improved expression of SERCA, maintenance of Ca^2+^ homeostasis within MAMs	[127]
Troglitazone	Sprague–Dawley rats	Reduced ACS activity in the liver MAM component and the peroxisome component	[128, 129]

Risk factors for NAFLD include obesity, T2DM, and metabolic syndrome, and one of the common features of these diseases is IR. Therefore, islet cell sensitizers are typically used to treat NAFLD [130]. Metformin improves hepatic and peripheral IR by reducing glucose absorption, hepatic gluconeogenesis, and FA synthesis and increasing β-oxidation. Studies have shown that CypD-KO mice treated with metformin for 4 weeks show improved insulin sensitivity and hepatic neovascularization and, more importantly, increased MAM levels in the liver [94]. Therefore, metformin may increase islet cell sensitivity by restoring MAM levels in the livers of mice. Studies have confirmed that metformin improves insulin sensitivity and cholesterol and aminotransferase levels in patients with NAFLD [131]. Metformin may be more appropriate as a component of a combination treatment regimen for patients with NAFLD.

Sulforaphane (SFN), which has been reported to act similarly to metformin, can improve disrupted ER–mitochondria interactions. Based on the results of one study, SFN enhances islet cell sensitivity to some extent by regulating MAMs, thus improving liver glucose tolerance, which can reduce liver lipid synthesis and reverse NAFLD [125]. The levels of the MAM-related proteins VDAC1, CypD and PACS2 in the livers of mice fed an high-fat and high-glucose diet (HFHGD) have been found to be significantly reduced, whereas Mfn2 levels are increased and IP3R1 levels are unchanged. In addition, the basal phosphorylation of PKB is increased in HFHSD-fed mice. In addition, rosiglitazone intervention restores CypD and PACS2 expression and PKB phosphorylation levels [94]. ACS4 is present in fractions identified to contain MAMs, and ACSL4 is linked to TAG synthesis. Troglitazone, a specific inhibitor of ACSL4, reduces acetyl-CoA synthetase activity in the liver MAM component by 30–45% and ACS activity in the peroxisome component by approximately 30% [128]. Data analyses have shown that thiazolidinedione has a good therapeutic effect on NASH in subjects with and without diabetes [129]. However, the detailed mechanisms by which these drugs affect liver histology have not been fully confirmed. Therefore, identifying drugs that target MAMs will be a crucial strategy for developing NAFLD treatments.

At present, most drugs targeting MAMs show certain effects in the treatment of NAFLD, but they are usually not used specifically for NAFLD. Although they target MAMs, these drugs, which fall within a single class, have limited usefulness for reversal of NAFLD due to the complex structures of MAMs and the complex pathogenesis of NAFLD. Clinically, multiple drugs are commonly used to treat NAFLD. Therefore, it is necessary to explore new drugs that target MAMs and to use them safely and effectively.

## Conclusion

This review highlights the main pathways of liver fat accumulation, including increased FA uptake and DNL and decreased FA β-oxidation and VLDL secretion. In hepatocytes, disruption of glycolipid metabolism is perceived by the ER and mitochondria, and certain levels of ERS and oxidative stress alleviate mild disorders. When these stresses exceed the regulatory limits, a series of signalling pathways (associated with the UPR, Ca^2+^ disturbances, and excess ROS production) lead to the development and progression of NAFLD. This review also summarizes the relevant evidence and reveals that mitochondrial, ER, and MAM integrity play crucial roles in the development of NAFLD. First, MAM integrity guarantees the exchange of Ca^2+^, ROS, and lipids between the ER and mitochondria, and a certain link between MAM-mediated Ca^2+^ stabilization and lipid and ROS exchange has been identified. Once Ca^2+^ homeostasis is disturbed, the UPR within the ER is induced, leading to the generation of ROS and the entry of ROS into the mitochondria. On the other hand, oxidative stress also leads to ROS production and crosstalk. ERS, ROS, and oxidative stress induction cause hepatic fat accumulation, steatosis, and further development of NASH through the induction of hepatic IR, apoptosis, inflammation, and mitochondrial dysfunction. MAMs not only serve as links between the ER and mitochondrial structures but also play irreplaceable roles in maintaining the balance between the ER and mitochondria.

To maintain the integrity of related proteins on MAMs, we must also ensure that MAMs have the appropriate spacing. Normal spacing is a key prerequisite for the functions of various proteins or signalling molecules on MAMs. Mfn2 is one of the proteins that is physically connected with the structures of MAMs. Mfn2 molecules on the OMM and the ER membrane can form dimers to bring the two organelles closer together. NAFLD caused by MAM integrity damage can be alleviated and inhibited by overexpression of Mfn2, which suggests that Mfn2 is a key drug target [122]. To ensure that the physical effects on MAMs are complete, some functional proteins located on MAMs can be further targeted to achieve the desired therapeutic effects. Research on MAM-related Ca^2+^ channels, the ERS signalling pathway and the oxidative stress signalling pathway has also yielded important breakthroughs in NAFLD research and drug development. This review discussed the crucial roles of these components and processes in the development of NAFLD. For clinical drug treatment, drugs targeting MAMs should be developed, and safe and effective combinations of drugs should be used to repair the integrity of MAMs in order to ensure the normal functioning of the ER and mitochondria in cells. The current research on the roles of MAMs in the pathogenesis of NAFLD is biased towards regulation of certain targets. However, the molecular mechanisms of the main pathogeneses of NAFLD differ among different stages or different types of NAFLD. Therefore, more research on the molecular mechanisms of NAFLD pathogenesis are needed to confirm the statuses of MAMs.

Here, we propose that MAMs act as bridges linking mitochondrial oxidative stress and ERS. Through interventions targeting MAMs, both stress responses may be simultaneously improved, thereby reversing NAFLD. The main challenges faced by researchers in the study of hepatocyte MAMs in NAFLD patients are as follows: 1) MAMs have complex structures and do not form true physical connections, rendering their main structures difficult to determine; 2) MAMs have strict requirements for spatial distance; 3) MAM spatial distance is regulated by a variety of proteins in a complicated manner; and 4) the contributions of some functional proteins located on MAMs are unclear, and there have been few reports on the subject. Certain drugs cannot specifically act on these contact points because of the complexity and dynamic natures of MAM structures, which has become a major obstacle to the prevention and treatment of NAFLD using strategies targeting MAMs. However, from another perspective, alteration of the expression of genes encoding some MAM-specific proteins and related upstream or downstream molecules has become a potential therapeutic strategy.

## Data Availability

Not applicable.

## Abbreviations

ACC: Acetyl-CoA carboxylase
ACL: ATP citrate lyase
ACS4: Acyl-CoA synthetase 4
ApoB: Apolipoprotein B
ATF6: Activating transcription factor 6
BAP31: B-cell-receptor-associated protein 31
BiP: Binding immunoglobulin protein
CHOP: C/EBP homologous protein
ChREBP: Carbohydrate response element binding protein
CPT: Carnitine palmitoyltransferase
CXCR3: C-X-C motif chemokine receptor 3
DAG: Diacylglycerol
DGAT: Diacylglycerol acyltransferase
DHC: Dihydroceramide
DHS: Dihydrosphingosine
DNL: De novo lipogenesis
ERO1: Endoplasmic reticulum oxidoreductin-1
ERS: Endoplasmic reticulum stress
FABP: Fatty acid binding protein
FACL4: Fatty acid-CoA ligase 4
FATP: Fatty acid transporter protein
FFAs: Free fatty acids
FxR: Farnesoid X receptor
G3P: Glyceraldehyde 3-phosphate
GADD34: Growth arrest and DNA damage gene
GPAT: Glycerol-phosphate acyl transferase
GRP78: Glucose-regulated protein 78
HCC: Hepatocellular carcinoma
HFHC: High-fat, high-carbohydrate
IP3R: Inositol 1,4,5-triphosphate receptor
IR: Insulin resistance
IRE1: Inositol-requiring enzyme 1
LBS: Lipid bilayer stress
LPA: Lysophosphatidic acid
LPAAT: Lysophosphatidic acid acyltransferase
LXR: Liver X receptor
MAMs: Mitochondria-associated membranes
MAT: Malonyl/acetyltransferase
MCD: Malonyl-CoA decarboxylase
MCU: Mitochondrial Ca2+ unidirectional transporter
MEFs: Mouse embryonic fibroblasts
MERCs: Mitochondria-ER contact sites
Mfn1/2: Mitofusin 1/2
MMO: Microsomal monooxygenase
mTORC2: Mammalian target of rapamycin complex 2
MUFAs: Monosaturated fatty acids
NAFLD: Nonalcoholic fatty liver disease
Nrf2: Nuclear factor E2-related factor 2
OMM: Mitochondrial membrane
ORP5 /8: Oxysterol-binding protein -related proteins5/8
p66Shc: 66-kilodalton isoform of the growth factor adaptor Shc protein
PA: Phosphatidic acid
PACS2: Phosphofurin acidic cluster sorting protein 2
PAP: Phosphatidic acid phosphorylase
PC: Phosphatidylcholine
Pcyt1a: Phosphate cytidylyltransferase 1, choline, alpha
PDI1: Protein disulfide isomerase
PE: Phosphatidyl ethanolamine
PEMT: Phosphatidylethanolamine N-methyltransferase
PERK: Protein kinase RNA-like ER kinase
PGC-1α: Peroxisome proliferator-activated receptor gamma coactivator-1α
PP2A: Pentose phosphate-2A
PPARα: Peroxisome proliferator-activated receptor-alpha
PS: Phosphatidylserine
PSD: Phosphatidylserine decarboxylase
PSS1/2: Phosphatidylserine synthetase 1 and 2
PTEN: Phosphatase and tensin homolog
ROS: Reactive oxygen species
SCD1: Stearoyl-CoA desaturase-1
SERCA: Sarco/endoplasmic reticulum Ca^2+^ −ATPase
SFAs: Saturated fatty acids
SFN: Sulforaphane
SREBP-1c: Sterol regulatory element binding protein 1c
T2DM: Type 2 diabetes mellitus
TCA: Tricarboxylic acid
TD: Transmembrane domain
TE: Thioesterase
TG: Triglyceride
TRPP2: Transient receptor potential protein 2
VDAC: Voltage-dependent anion channel
VLDLs: Very low-density lipoproteins
WAT: White adipose tissue
